# Solid Solutions of Rare Earth Cations in Mesoporous Anatase Beads and Their Performances in Dye-Sensitized Solar Cells

**DOI:** 10.1038/srep16785

**Published:** 2015-11-18

**Authors:** Carmen Cavallo, Alberto Salleo, Daniele Gozzi, Francesco Di Pascasio, Simone Quaranta, Riccardo Panetta, Alessandro Latini

**Affiliations:** 1 Dipartimento di Chimica, Università degli Studi di Roma “*La Sapienza*”, Piazzale Aldo Moro 5, 00185 Roma, Italy; 2 Geballe Laboratory for Advanced Materials, Department of Materials Science and Engineering, 239 McCullough Building, Stanford University, Stanford, CA 94305, USA; 3 Faculty of Science, University of Ontario Institute of Technology, 2000 Simcoe Street North, Oshawa, Ontario, Canada, L1H 7L7

## Abstract

Solid solutions of the rare earth (RE) cations Pr^3+^, Nd^3+^, Sm^3+^, Gd^3+^, Er^3+^ and Yb^3+^ in anatase TiO_2_ have been synthesized as mesoporous beads in the concentration range 0.1–0.3% of metal atoms. The solid solutions were have been characterized by XRD, SEM, diffuse reflectance UV-Vis spectroscopy, BET and BJH surface analysis. All the solid solutions possess high specific surface areas, up to more than 100 m^2^/g. The amount of adsorbed dye in each photoanode has been determined spectrophotometrically. All the samples were tested as photoanodes in dye-sensitized solar cells (DSSCs) using N719 as dye and a nonvolatile, benzonitrile based electrolyte. All the cells were have been tested by conversion efficiency (*J*–*V*), quantum efficiency (IPCE), electrochemical impedance spectroscopy (EIS) and dark current measurements. While lighter RE cations (Pr^3+^, Nd^3+^) limit the performance of DSSCs compared to pure anatase mesoporous beads, cations from Sm^3+^ onwards enhance the performance of the devices. A maximum conversion efficiency of 8.7% for Er^3+^ at a concentration of 0.2% has been achieved. This is a remarkable efficiency value for a DSSC employing N719 dye without co-adsorbents and a nonvolatile electrolyte. For each RE cation the maximum performances are obtained for a concentration of 0.2% metal atoms.

Anatase has been and still is one the most studied simple oxides by the worldwide scientific community. This interest is mainly due to its exceptional photochemical properties which make it the standard material for photocatalysis experiments and the best performing photoanode material in DSSCs and lead halide perovskite sensitized solar cells (PSSC)^1^. In addition, its lack of toxicity, ease of preparation with an exceptionally wide range of morphologies^2^,^3^,^4^, and low cost (titanium is the ninth most abundant element in Earth’s crust^5^) further justify the interest on this exceptional material.

Furthermore, anatase is used in sunscreen formulations and self-cleaning paints and coatings because of its photochemical properties.

Finally, low energetic and financial production costs makes anatase an attractive material for photovoltaic applications. A large number of papers on DSSCs and PSSCs has been published since their discovery. For example, 2494 papers on DSSCs and 397 papers on PSSCs have been published during the year 2014, only considering scientific publications written in English and excluding patents (source: SciFinder). Both technologies have their pros and cons. DSSCs are a quite mature technology, with 24 years of developments since the first publication^4^, and their introduction on the market seems imminent. They probably constitute the cheapest photovoltaic technology available today. Besides, DSSCs’ capability of working very well in low and diffuse light conditions partially compensates for their relatively low conversion efficiencies. Unfortunately, their efficiency improvement during the last 24 years has been quite modest (from 7 to 13%)^6^,^7^,^8^. On the other hand, PSSCs showed an amazing development and their conversion efficiency raised up from less than 4% when they were described for the first time in 2009^9^ to more than 20% in 2015^7^. But PSSCs suffer from serious stability issues^10^ and in some cases, their hysteretic behavior may lead to wrong evaluation of their performances^11^.

An improvement of the performances of both DSSCs and PSSCs may be obtained quite simply by “tuning” the physico-chemical properties of anatase.

Such as optimization has been widely investigated in literature for DSSCs, but the most of the work has been focused on the morphology optimization of anatase or on the study of other semiconducting oxides such as ZnO or SnO_2_^12^. The number of papers dedicated to systematic studies of the effect of heteroatoms and their concentration in the anatase lattice on the performances of DSSCs are far less numerous. For instance, among the papers of Grätzel and coworkers on DSSCs, only two are dedicated to this topic (one to the effect of Ga^3+^ and Y^3+^ in mesoporous anatase and one to the effect of Nb^5+^ in nanocrystalline anatase for DSSCs’ photoanodes)^13^,^14^. Anatase predisposition to be doped with both cations and anions (probably because of its open crystal structure in comparison with TiO_2_ more thermodynamically stable polymorph, rutile) may lead to substantial improvements in the performances of photoanodes for both DSSCs and PSSCs. Our research group has been involved in the study of new materials and electrolytes for DSSCs for several years, and interesting results have been achieved and published^15^,^16^,^17^,^18^. One of our papers^16^ has shown the beneficial effect of Sc^3+^ doping of mesoporous anatase beads on the performances of DSSCs. This result has encouraged a systematic study of the effect of RE cations on anatase beads for the same use. In addition, the development of a new, nonvolatile electrolyte has allowed us to achieve a remarkable efficiency value of 8.1% with a conventional, two-layered (transparent layer + scattering layer) photoanode made of commercial titania sensitized with N719 dye^17^. The aforementioned results have stimulated our research to fully exploit the potential of our new electrolytic composition in conjunction with a better performing photoanode, that is also simpler to be realized, being the functions of the transparent and the scattering layers joined in one layer when anatase beads are used^19^.

Because of their unique properties, rare earth ions lend themselves to a systematic study on anatase electronic properties modulation through the combined effects of both ionic size and dopant concentration with the key opportunity of keeping the same valence state. In fact, they have nearly identical chemical properties (with the notable exceptions of Ce and Eu), they all have the trivalent oxidation state as the most stable one (except for Ce), but their ionic radius (for trivalent cations) decreases monotonically along the series due to the lanthanide contraction effect. Hence, a systematic work of synthesis, characterization and test of DSSC photoanodes containing mesoporous anatase beads doped with RE cations was carried out in our labs. Solid solutions were prepared with all the RE possessing commercially available alkoxides, i.e. Pr, Nd, Sm, Gd, Er, and Yb with concentrations 0.1, 0.2 and 0.3% of total metal atoms.

## Results and Discussion

**Figure 1:**
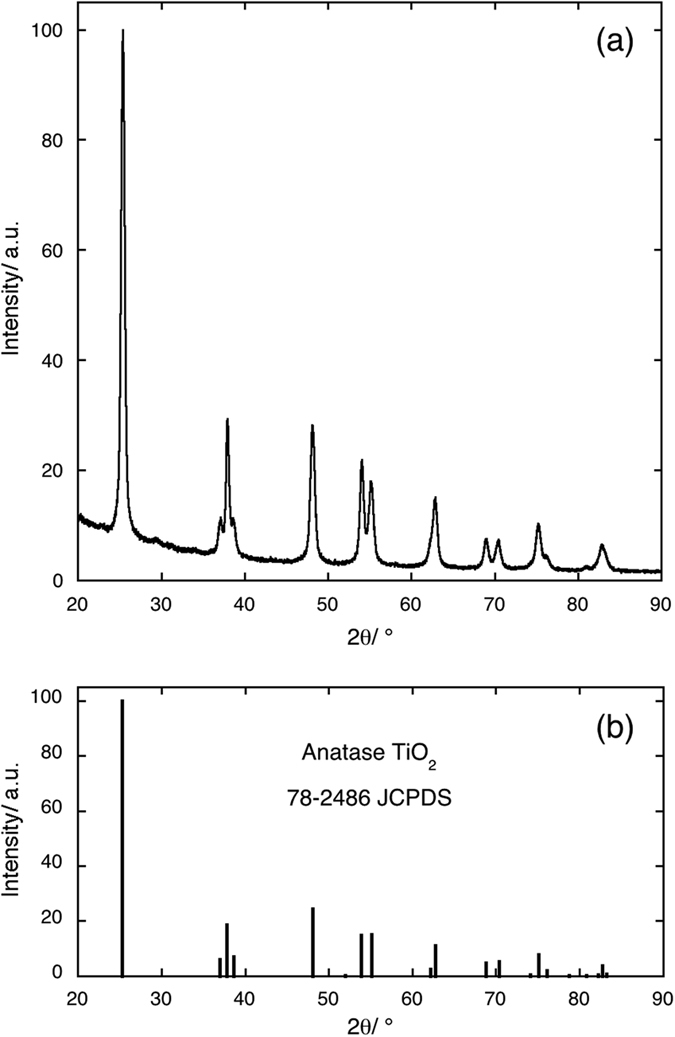
XRD pattern of the solid solution containing Er at a concentration of 0.2% total metal atoms (panel a) and reference pattern of anatase (panel b).

**Figure 2:**
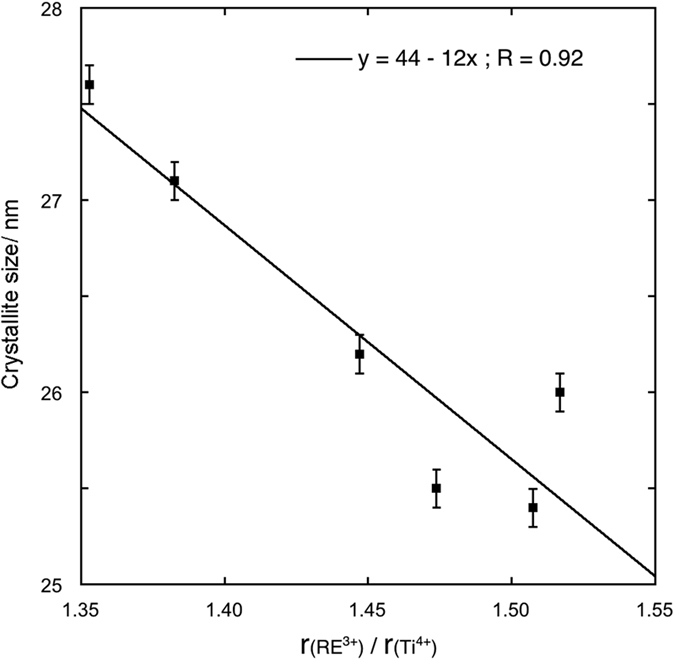
Fit of the crystallite size values obtained from the Rietveld refinement procedure for solid solutions containing RE cations at a concentration of 0.2% total metal atoms versus the radius ratio RE^3+^/Ti^4+^.

The X-ray diffraction patterns of all samples show only the presence of the reflections due to the anatase phase of TiO_2_. In Fig. 1, panel a, one pattern is given as an example (TiO_2_: Er^3+^ 0.2%), while in panel b the positions and the relative intensities of the reference pattern^20^ are reported. The results of the Rietveld refinement of the experimental patterns (unit cell parameters and mean crystallite size) are given in Table 1. The dopant nature and its concentration have no substantial effect on the unit cell parameters. This is not surprising, if the small concentration of RE cations in the lattice is considered. Obviously, from powder diffraction patterns no information concerning the positioning of RE cations in the anatase lattice, i.e. substitutional or interstitial, can be inferred. Our previous work on Sc^3+^-containing anatase solid solutions^16^, in which the position of Sc cation was determined by EXAFS spectroscopy, showed that Sc was in substitutional sites up to very high Sc concentrations (10% metal atoms). Hence, we expect to find the same situation in the case of RE cations because of the strong similarity they have with Sc cation. The substitutional hypothesis is also supported by other works for trivalent cations in general^13^. It is worth noting that plotting the crystallite mean size vs. the RE^3+^/Ti^4+^ radius ratio^21^ for the best performing compositions, i.e. 0.2% metal atoms for all the RE under consideration, a satisfactory linear fit with negative slope can be obtained (Fig. 2). A possible explanation of this trend could lie in the Fajans’ rules^22^. Cations of equal charge form compounds with less ionic character as the ionic radius decreases, and their compounds with basic anions (hydroxides^23^, alkoxides, etc) become less basic with decreasing ionic radius. Thus lighter (and consequently bigger) RE cations give more basic alkoxides which may catalyse the hydrolysis reaction and producing more nuclei in the initial stages of the reaction, resulting in smaller particles.

**Figure 3:**
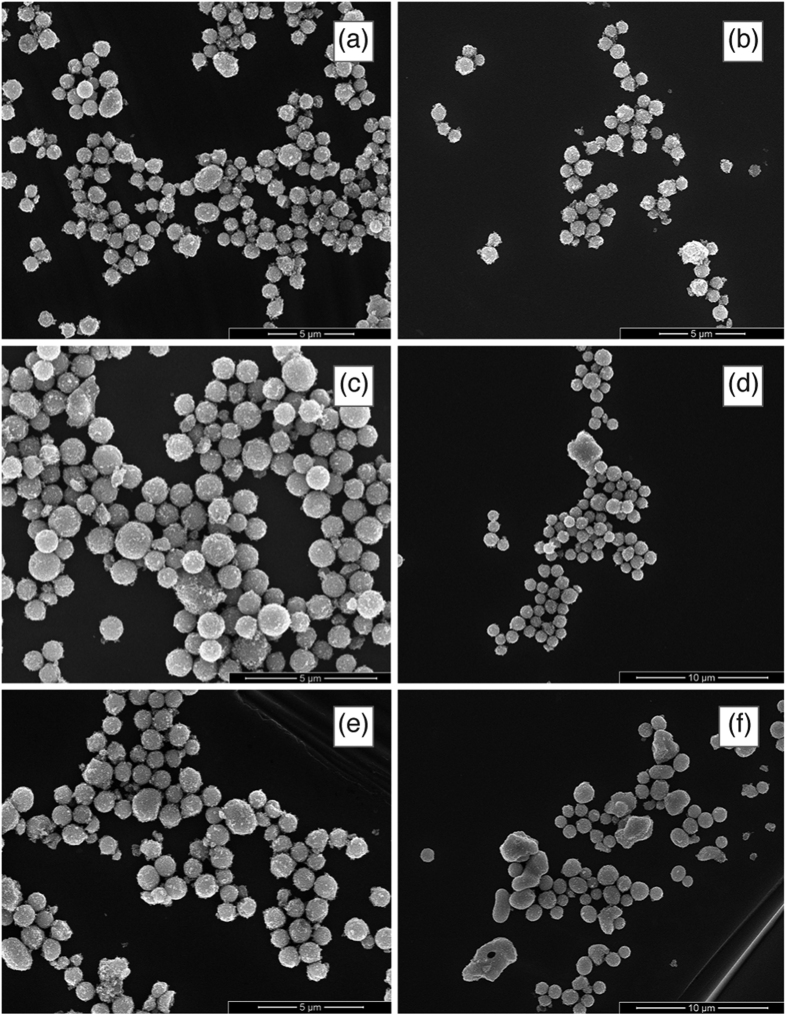
SEM micrographs of solid solution beads (RE cations at a concentration of 0.2% total metal atoms) at low magnification.

**Figure 4:**
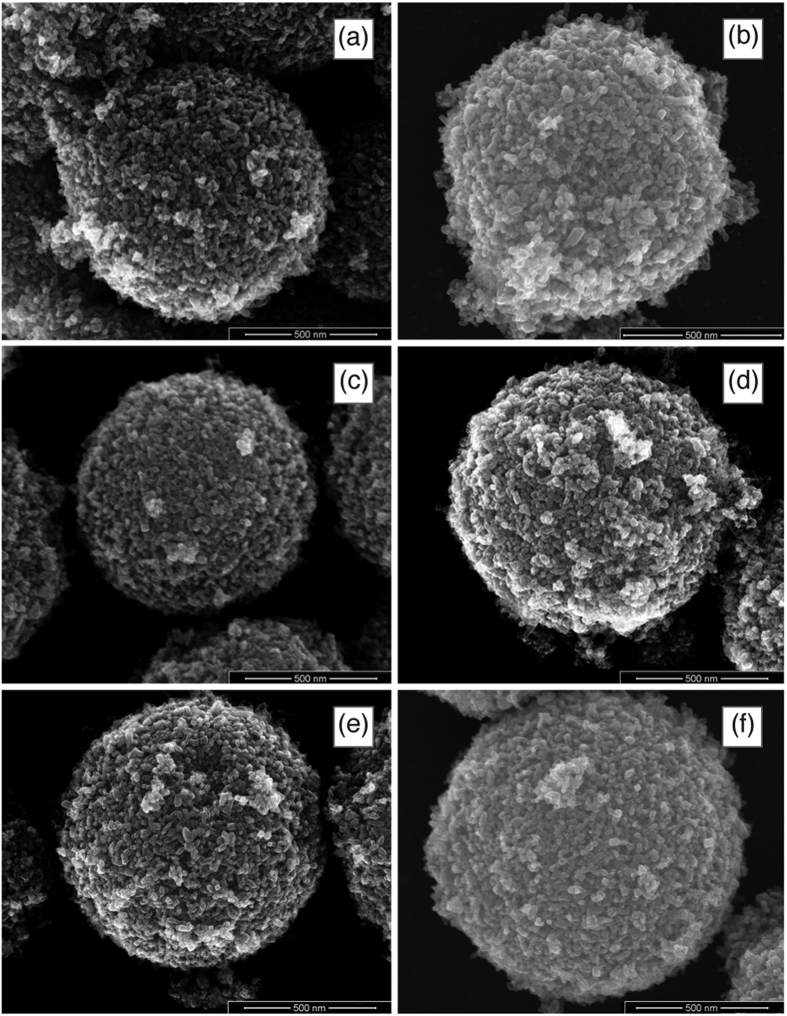
SEM micrographs of solid solution beads (RE cations at a concentration of 0.2% total metal atoms) at high magnification.

SEM micrographs for the 0.2% metal atoms samples are given in Figs 3 and 4 for Pr, Nd, Sm, Gd, Er, Yb in panel a, b, c, d, e, f respectively. No evident differences are detectable among the various samples and samples with different RE cations. Samples with different RE concentrations are practically identical. The beads appear as nearly monodisperse, submicrometric porous spheroidal conglomerates of crystallites with dimensions in agreement with those determined by XRD.

BET-BJH analyses confirm the results of the structural and morphological investigations.

In Table 2 a summary of the results of BET specific surface area values for 0.2% samples are reported, and also in this case, the results for the other compositions are analogous. All the samples possess quite high specific surface area values, ranging from about 60 to 100 m^2^/g. The mean pore size diameter calculated by the BJH method is around 15–16 nm for all the samples.

**Figure 5:**
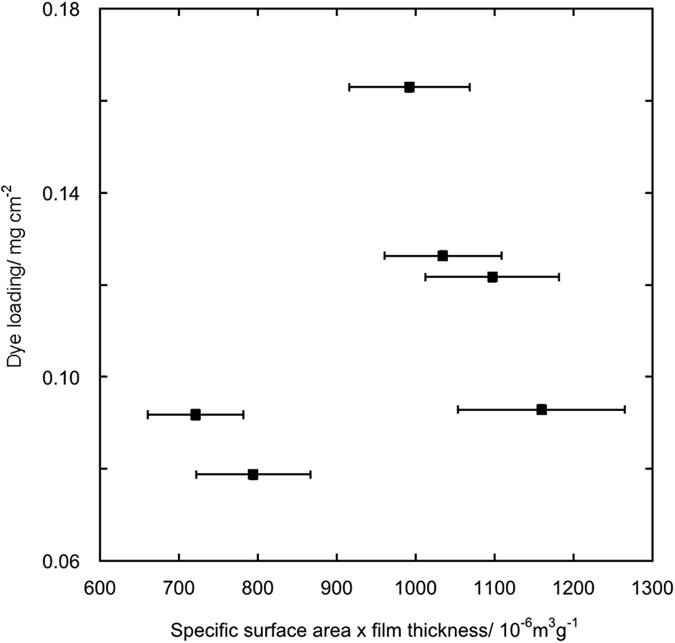
Dye loading values for the photoanodes made with 0.2% RE cations solid solutions versus the product of solid solution specific surface area and photoanode thickness.

**Figure 6:**
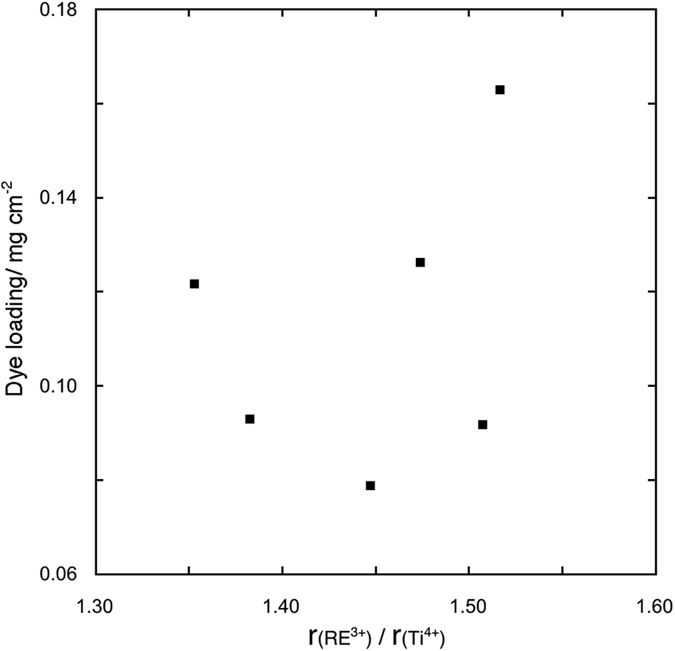
Dye loading values for the photoanodes made with 0.2% RE cations solid solutions versus the RE^3+^/Ti^4+^ radius ratio.

In Table 3 the photoanode thickness and the dye loading values are presented. No evident correlation is present between the amount of the adsorbed dye and the specific surface area, also taking into account the film thickness (assuming a constant film density). Besides, no correlation is present between the dye loading and the radius ratios (Figs 5 and 6, respectively). These lacks of correlation, together with the lack of correlation between the open circuit voltage under illumination (*Voc*) values and the radius ratios (see later), implies that the introduction of RE cations in the anatase lattice modifies the surface chemistry of the solid solutions in a quite unpredictable way. In fact, rare earth ions modify both the chemisorption equilibrium of the dye with the surface and the equilibrium of adsorption of electrolyte additives as TBP, guanidinium cation and Li^+^; consequently the *Voc* values are sensibly affected by blocking effect and/or shifting the TiO_2_ conduction band edge^24^,^25^.

The results obtained from band gap measurements and from the *J*–*V* curves under AM 1.5 G simulated sunlight, i.e. *η*, short circuit current density *Jsc*, *Voc*, fill factor *FF*, series resistance *Rs*, and in dark, i.e. saturation current *J*_*0*_, ideality factor *m* and calculated *Voc* are presented in Table 4. The data obtained from the *J*–*V* curves in dark were have been calculated by using the modified diode equations:


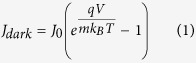



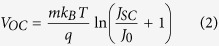


where *q* is the elementary charge and *k*_*B*_ the Boltzmann constant. The terms *J*_*0*_ and *m* have been extracted from the high voltage part of the dark current curves where eqn. 1 can be approximated as:


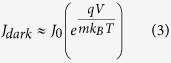


The linear fit of the ln *J*_*dark*_ vs. *V* allows to extrapolate *J*_*0*_ and *m* from the intercept and the slope, respectively, considering that:


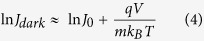


**Figure 7:**
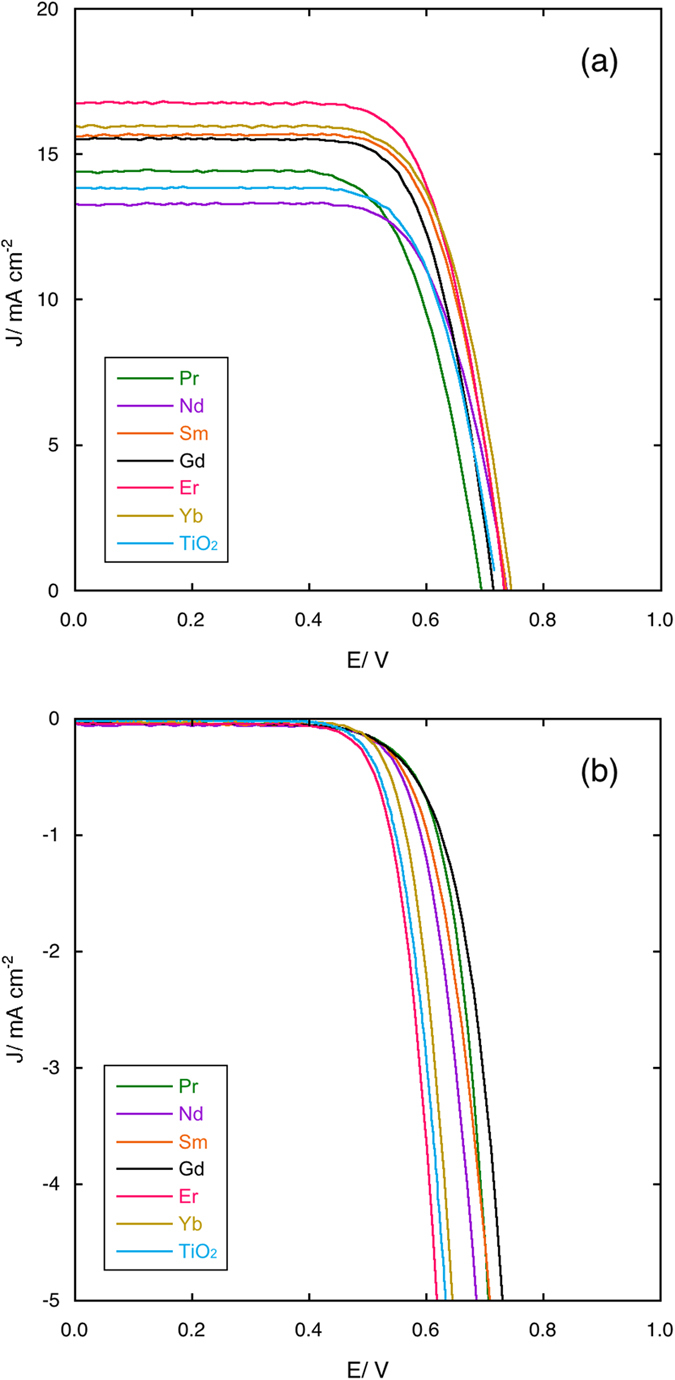
*J*–*V* curves under light (panel a) and in dark (panel b) for cells assembled with photoanodes with 0.2% RE cations solid solutions.

For comparison purposes, the data of a DSSC made with mesoporous beads of pure TiO_2_ are presented. In Fig. 7 the *J*–*V* curves under illumination (panel a) and in dark (panel b) for 0.2% samples are shown.

Two facts immediately stand out:

- all the RE with the exception of Pr and Nd improve the performances of the devices;
- for all the RE the maximum performances are obtained for a concentration of RE cations of 0.2% metal atoms.

**Figure 8:**
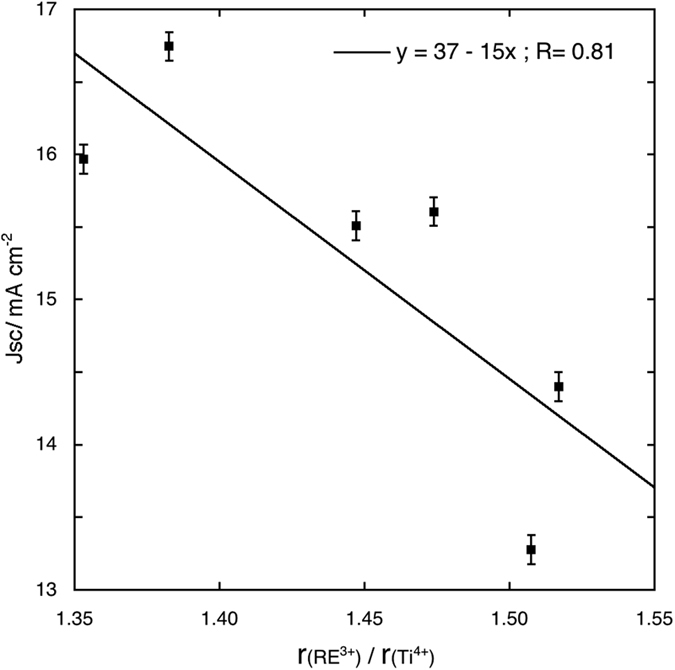
Fit of the *Jsc* values for cells assembled with photoanodes with 0.2% RE cations solid solutions versus the RE^3+^/Ti^4+^ radius ratio.

**Figure 9:**
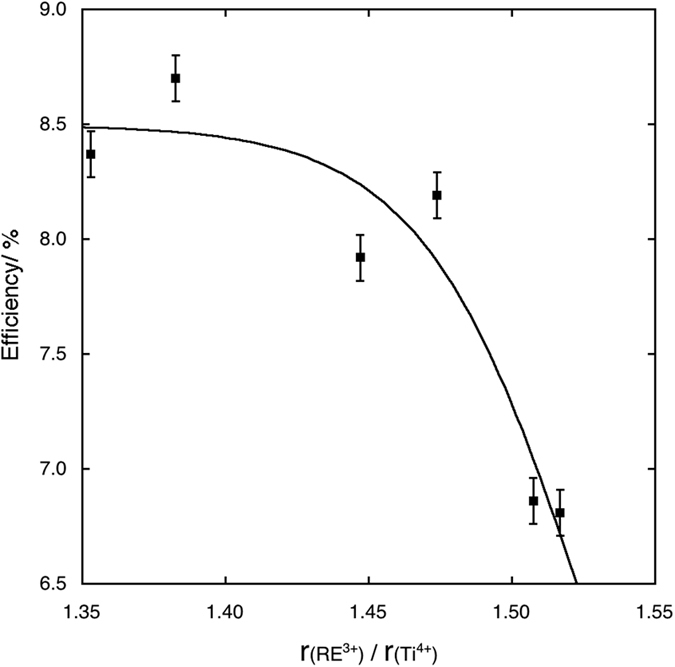
Asymptotic fit of the efficiency values of cells assembled with photoanodes with 0.2% RE cations solid solutions versus the RE^3+^/Ti^4+^ radius ratio.

A possible explanation of these phenomena may be found in the electron scattering caused by defects. The excessive distortion of the anatase lattice caused by the biggest cations may hinder electron transport and, in fact, the photoanodes made with Pr and Nd doped anatase present the lowest *Jsc* values. In Fig. 8, *Jsc* is plotted against the radius ratios for the 0.2% samples and its decreasing trend is quite apparent. The 0.2% optimal concentration is more difficult to explain, but may be a sort of “threshold” value, over which the defect scattering of electrons become so important that any possible beneficial effect would be overcome. A strong support to this hypothesis is the fact that also in the case of Sc^3+^ solid solutions, the best performances have been observed for the same concentration^16^. The plot of the *η* values vs. the radius ratios (Fig. 9) shows an asymptotic behaviour which can be fitted by an equation of the type:


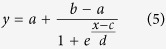


The highest *η* value (8.7%) has been obtained for the Er 0.2% sample,. which is a valuable result for a DSSC using a nonvolatile electrolyte and N719 dye without co-adsorbents and/or surface passivating agents. The efficiency value for the Yb 0.2% sample is only slightly lower (8.4%).

No correlation was found between the experimental values of *Voc* and those calculated by using eqn. 2 (Table 4). Probably the diode model is too simplistic to describe the behaviour of the DSSCs and an indirect proof of this is given by the very high values of the ideality factors. In fact, using a single parameter to describe all the deviations from the ideal diode model may result insufficient, especially when an electrolyte with a relatively low conductivity and high I_3_^−^ anion diffusion coefficient is employed^17^. The band gap values do not display any correlation with the dopant type and/or its concentration, probably because the concentration values are too low to have a significant effect, as in the case of unit cell parameters in XRD analysis.

**Figure 10:**
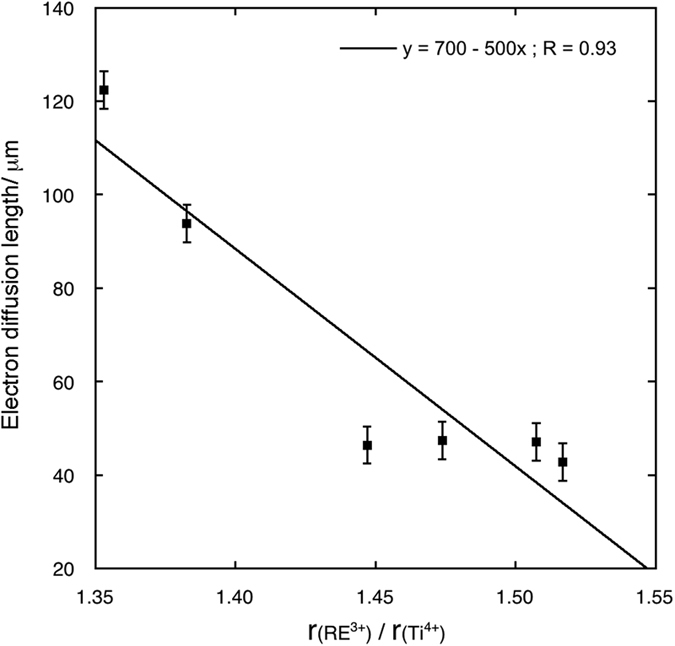
Fit of the electron diffusion length values for cells assembled with photoanodes with 0.2% RE cations solid solutions versus the RE^3+^/Ti^4+^ radius ratio.

**Figure 11:**
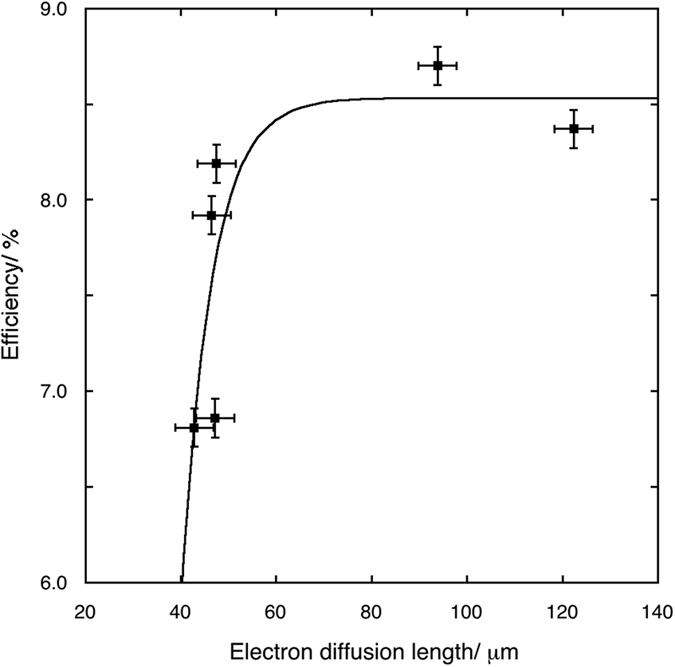
Asymptotic fit of the efficiency values of cells assembled with photoanodes with 0.2% RE cations solid solutions versus the electron diffusion length.

Electron diffusion lengths and charge collection efficiencies calculated from the fit of EIS spectra are reported in Table 5. Figures 10 and 11 show the trends of the electron diffusion length vs. the radius ratios and of the *η* values vs. electron diffusion length are shown, respectively for 0.2% samples. The electron diffusion length decreases with an approximately linear trend as the radius ratios increases, while the efficiency increases with an asymptotic law as the electron diffusion length increases. In this last case, the best fit of the values has been obtained with the equation type:


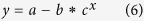


Both these results are in agreement with those obtained from the analyses of *J*–*V* curves under light and in dark.

In the case of the Er 0.2% and Yb 0.2% samples electron diffusion length and charge collection efficiency values apparently contradict the photovoltaic efficiency. In fact, the Er 0.2% sample shows higher photovoltaic performances compared to the Yb 0.2% despite possessing lower electron diffusion length and charge collection efficiency. These seemingly unexpected results can be justified by the higher series resistance value for the Yb 0.2% sample and by the following observations:

- the efficiency values are very similar, being their difference only slightly higher than the uncertainty on the values, and the same applies to the electron collection efficiency values;

- the ratio of electron diffusion length to film thickness is more significant than the diffusion length itself in determining the performances of the cells. Those ratios are practically identically for 0.2% Er and 0.2% Yb, if the uncertainties are taken into account.

**Figure 12:**
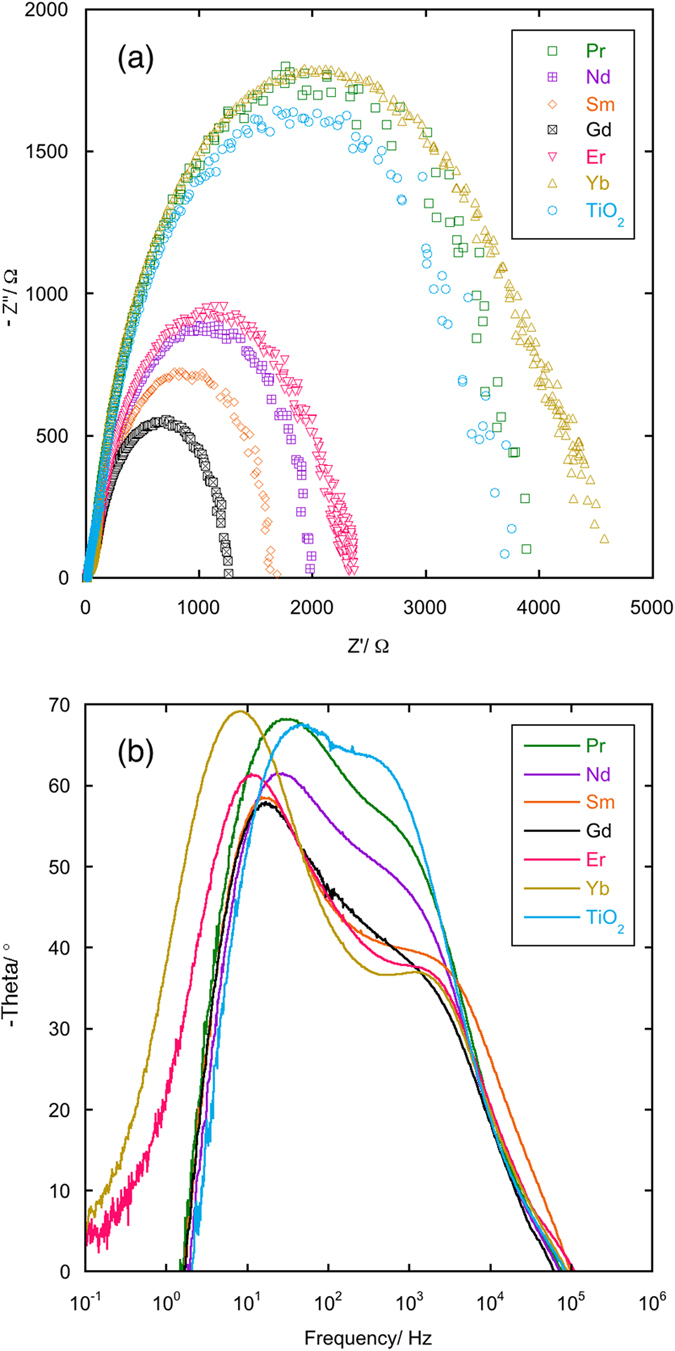
Nyquist plots (panel a) and Bode plots (panel b) for 0.2% RE cations solid solutions and pure TiO_2_.

From what observed and discussed until now, a fundamental question does not find answer, i.e. why do RE cations (heavier than Sm^3+^ and including it) improve the performances of anatase in the photoanodes of DSSC with respect to pure anatase? A possible explanation is that trivalent RE cations, at low concentration levels, suppress the natural oxygen defectivity of anatase^26^ which is responsible for electron trapping. In fact, Chandiran *et al.*^13^ experimentally observed a surprising substantial decrease of resistivity in Y^3+^-doped anatase at low dopant concentration (~0.1%) in comparison to pure TiO_2_, since they expected creation of more oxygen vacancies that would theoretically create a trapping effect with an opposite effect on resistivity. In Fig. 12 the Nyquist plots (panel a) and the Bode plots (panel b) for the 0.2% samples and pure TiO_2_ are reported. The suppression of the oxygen defectivity by RE cations is supported by the fact that the maximum frequency peak in the mid-frequency range of the phase Bode plot shifts to lower values for the samples containing rare earths ions with respect to pure TiO_2_. The shift is maximum for the best performing samples, i.e. Er 0.2% and Yb 0.2%. Similar features can be observed in the Nyquist plots: the recombination frequency (the maximum in the complex impedance graph) for the doped samples is lower than the one for pure TiO_2_. Consequently, all the RE doped cells have higher recombination lifetimes with respect to pure anatase. This explanation, coupled with the electron scattering by defects, would justify all the trends shown in Figs 8, 9, 10, 11.

**Figure 13:**
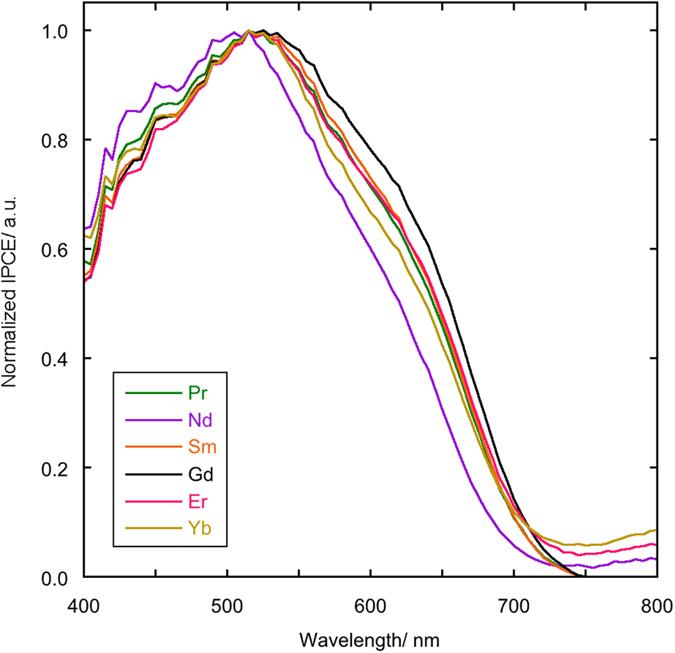
Normalized IPCE spectra of cells assembled with photoanodes with 0.2% RE cations solid solutions.

In Fig. 13 a comparison of normalized IPCE spectra are given for 0.2% samples. While samples containing Pr, Sm, Er and Yb all present very similar behaviours, those containing Nd and Gd are the ones that deviate most. The 0.2% Nd sample shows an enhanced response at lower wavelengths (<500 nm) and a depressed one at higher wavelengths. The 0.2% Gd sample shows a substantially enhanced response in comparison with the other samples at wavelengths >500 nm, and this may justify the fact that the cell has a high efficiency (7.9%) though it shows the second shortest electron diffusion length among the 0.2% samples after the Pr containing sample.

## Conclusions

Mesoporous beads of solid solution of anatase containing the rare earth cations Pr^3+^, Nd^3+^, Sm^3+^, Gd^3+^, Er^3+^ and Yb^3+^ with cation concentrations 0.1, 0.2 and 0.3% of metal atoms have been prepared and characterized structurally, morphologically and optically. The nature of the rare earth cation does not substantially affect the morphology of the beads, which presents similar average dimensions, surface area and porosity. Structurally, the rare earth concentration in the lattice seems too low to affect unit cell parameters, while an evident correlation in 0.2% samples between the rare earth cation size and crystallite average size, probably as a result of the Fajans’ rules, has been found. The band gap values of the solid solutions do not show significant deviations from that of pure anatase (3.2 eV).

The solid solutions have been used for the preparation of DSSCs’ photoanodes using N719 dye and a nonvolatile electrolyte. The type of cation and its concentration have a profound influence on the behavior of the device. While Pr and Nd suppress the performances of the DSSCs, all the others improve them, and the best performances are obtained for a RE concentration of 0.2% metal atoms.

The cells were characterized by *J*–*V* curves under light and in dark, EIS spectroscopy and IPCE measurements. The maximum efficiency has been obtained for the sample containing 0.2% Er metal atoms (8.7%).

The analysis of the experimental data and a survey of the available literature suggest that RE cations in the anatase lattice produce two conflicting effects that affect DSSC performances, i.e. defect scattering due to the lattice distortion and the suppression of natural oxygen defectivity, which reduces intra-gap trap states in anatase that affect the electron diffusion length. While for Pr^3+^ and Nd^3+^, being the biggest ions of the series, the first effect prevails, so decreasing the performances of the DSSC devices in comparison with pure TiO_2_, the opposite is true for heavier (and smaller) cations than Sm^3+^ and including it, with which a substantial increase of DSSC performances can be achieved.

## Methods

All the solid solution anatase beads have been prepared by a modification of a well established procedure of controlled hydrolysis of titanium tetraisopropoxide (TIP) in hydroethanolic solution of hexadecylamine (HDA) and KCl^27^, in which a proper amount of TIP is replaced by a corresponding amount of a RE isopropoxide, depending on the desired composition of the solid solution. All the autoclavation and thermal treatment procedures are the same as reported in literature^27^. To prepare DSSC photoanodes, ethylcellulose-terpineol based screen-printing pastes have been prepared using the beads and following the literature procedure^28^ to which another step was added, i.e. a thermal treatment of the paste at 80 °C for 4 h to increase its stability. The photoanodes have been printed on 10 Ω/◻ FTO glass slides (thickness 3 mm, XOP Fisica, Spain) previously cleaned with alkaline (NH_4_OH/H_2_O_2_) and acid (HCl/H_2_O_2_) RCA-processes and then treated with the standard 40 mM aqueous TiCl_4_ solution at 70 °C for 30 min^29^. A 34T polyester mesh has been used for the printing process on a manual screen printer (Mismatic, Italy) and, after film relaxation and drying at 125 °C for 6 min, the printing process has been repeated until a final thickness (after calcination) of 10–15 μm was obtained. The printed films have been subsequently calcined in air and then the TiCl_4_ treatment repeated^28^,^29^. DSSCs have been assembled after sensitizing the photoanodes with concentrated N719 solution^30^ by sealing it together a platinized 15 Ω/◻^31^ FTO glass slide (thickness 3 mm, XOP Fisica, Spain) acting as cathode. Also the glass slides used to prepare the cathodes have been cleaned using alkaline and acid RCA treatments before use. The sealing of the cells was accomplished by using 25 μm Surlyn gaskets subsequently melted at 110 °C. Once cooled to room temperature, the cells have been filled with the electrolyte through 1 mm hole later closed with 60 μm Surlyn gaskets covered with a thin glass slide and melted with a soldering iron tip. The leads have been soldered to the cells utilizing the the Cerasolzer CS246-150 soldering alloy and a MBR Electronics USS-9210 Ultrasonic Soldering System. Kynar PVDF 502-CUH-HC film has been used as anti-reflection and UV blocking layer (<400 nm) on the photoanode side and was kindly given as free sample by Arkema Inc. The electrolyte is composed by benzonitrile as solvent and contains: 0.6 M 1-ethyl-3-methylimidazolium iodide (EMII); 0.5 M 4-tert-butylpyridine (TBP); 0.1 M guanidinium thiocyanate (GuSCN); 0.1 M LiI and 0.03 M I_2_.

A minimum of 3 cells for each solid solution composition have been assembled to ensure reproducibility.

Titanium (IV) isopropoxide (TIP) (Vertec, 97+%), lithium iodide (ultra dry, 99.999%), iodine (99.9985%), erbium isopropoxide and ytterbium isopropoxide have been purchased from Alfa Aesar. Praseodymium, neodymium, samarium and gadolinium isopropoxides have been purchased from Strem Chemicals. Titanium (IV) chloride (99.9%), TBP (96%), acetonitrile (absolute, ≥99.5%, over molecular sieves), di-tetrabutylammonium cis-bis (isothiocyanato) bis (2,2′-bipyridyl-4,4′-dicarboxylato) ruthenium(II) (N719 dye, 95%), tetrabutylammonium hydroxide 30-hydrate, ethanol (absolute, ≥99.8%), hydrogen peroxide solution (34.5–36.5%), acetic acid (99–100%), ammonium hydroxide solution (30–33%), hydrochloric acid (≥37%), anhydrous terpineol, 5–15 mPa·s ethyl cellulose (48.0–49.5% w/w ethoxyl basis), 30–70 mPa·s ethyl cellulose (48.0–49.5% w/w ethoxyl basis), 1-hexadecylamine (HDA) (technical, 90%), GuSCN (≥99%), and benzonitrile (99.9%) have been purchased from Sigma Aldrich. Hydrogen hexachloroplatinate (IV) hydrate (40% Pt by weight) has been purchased from Chempur. EMII (>98%) was purchased from Iolitec.

The structural analysis of the samples has been performed by X-ray powder diffraction using a Panalytical X’Pert Pro MPD diffractometer (Cu Kα radiation, λ = 1.54184 Å) equipped with a X’Celerator ultrafast RTMS detector. An angular range 10–90° in 2θ has been explored. The angular resolution (in 2θ) was 0.001°. A 0.04 rad soller slit, a 1° divergence slit and a 20 mm mask have been used on the incident beam path, while a 6.6 mm anti-scatter slit and a 0.04 rad collimator have been used on the diffracted beam path. The Rietveld analysis of the diffraction patterns has been performed using the MAUD software package^32^, obtaining the values of the unit cell axes, volume and the mean crystallite size.

The geometrical area of the photoanodes has been measured according to literature procedure^28^.

Morphological analysis of the samples has been performed by a FEI Magellan XHR scanning electron microscope.

An A.P.E. Research (Italy) MAP3D-25 stylus profilometer has been used to measure the thickness of photoanodes.

A Shimadzu (Japan) UV2600 UV-Vis spectrophotometer has been used for the quantification of the dye loading on each photoanode (by desorbing the dye after the sensitization with aqueous NaOH 0.02 M solution) and for the determination of the band gap value of the solid solutions. In this latter case, a ISR-2600 Plus integrating sphere has been connected to the spectrophotometer for the purpose, working in reflectance condition. As reflectance reference, BaSO_4_ powder has been used. The band gap was calculated fitting the data with the Tauc’s plot^33^ assuming an allowed indirect interband transition^34^.

Specific surface area and pore size distribution measurements have been carried out using a Quantachrome Autosorb iQ gas sorption analyzer.

The 1286 Electrochemical Interface coupled with a 1260 Frequency Response Analyzer from Solartron Analytical, U.K., using the Full Combo ZPLOT/CorrWare software by Scribner Associates Inc., USA, has been used to collect the *J*–*V* curves under illumination and in dark and the electrochemical impedance spectra (EIS). The *J*–*V* curves under illumination has been acquired in potential stair-step mode with a step size of 10 mV and a step time of 1 s. Data acquisition has been repeated until a reproducible behaviour was observed. An Asahi Spectra (Japan) HAL-320, AM 1.5 G class A solar simulator has been used for determining the conversion efficiency. A calibrated Asahi Spectra Sun Checker has been used to check the intensity of the radiation to be within ± 1% of 1 sun.

Dark current curves have been collected with the same settings of those ones collected under illumination.

The EIS spectra were obtained under 1 sun illumination provided by a white light led with a DC bias of ~0.5 V. The spectra have been fitted using the ZView software adopting the transmission line model^35^.

The incident photon to current conversion efficiency (IPCE) has been measured in DC mode with a white light bias by a custom-made apparatus^16^,^36^. The white light bias was given by a white light led coupled with an optical fiber guide. All the measurements has been performed in the wavelength range 400−800 nm with a scan interval of 5 nm.

## Additional Information

**How to cite this article**: Cavallo, C. *et al.* Solid Solutions of Rare Earth Cations in Mesoporous Anatase Beads and Their Performances in Dye-Sensitized Solar Cells. *Sci. Rep.* **5**, 16785; doi: 10.1038/srep16785 (2015).

## Figures and Tables

**Table 1:** Rietveld refinement results of the X-ray diffraction patterns of the samples under study. Unit cell axes and mean crystallite size values are given.

Sample	*a*/Å	*c*/Å	D/nm
Pr 0.1%	3.7832 ± 0.0003	9.4937 ± 0.0008	26.5 ± 0.2
Pr 0.2%	3.7834 ± 0.0002	9.4945 ± 0.0005	26.0 ± 0.1
Pr 0.3%	3.7833 ± 0.0002	9.4943 ± 0.0005	26.9 ± 0.1
Nd 0.1%	3.7834 ± 0.0002	9.4942 ± 0.0005	25.8 ± 0.2
Nd 0.2%	3.7843 ± 0.0002	9.4977 ± 0.0005	25.4 ± 0.1
Nd 0.3%	3.7848 ± 0.0002	9.4994 ± 0.0005	25.5 ± 0.1
Sm 0.1%	3.7835 ± 0.0002	9.4963 ± 0.0005	27.1 ± 0.2
Sm 0.2%	3.7835 ± 0.0003	9.4953 ± 0.0008	25.5 ± 0.2
Sm 0.3%	3.7835 ± 0.0005	9.496 ± 0.001	26.3 ± 0.3
Gd 0.1%	3.7832 ± 0.0002	9.4954 ± 0.0006	26.5 ± 0.2
Gd 0.2%	3.7829 ± 0.0002	9.5002 ± 0.0006	26.2 ± 0.1
Gd 0.3%	3.7838 ± 0.0002	9.4964 ± 0.0005	26.8 ± 0.1
Er 0.1%	3.7842 ± 0.0001	9.4972 ± 0.0004	27.0 ± 0.1
Er 0.2%	3.7842 ± 0.0001	9.4990 ± 0.0006	27.1 ± 0.2
Er 0.3%	3.7843 ± 0.0002	9.4975 ± 0.0006	25.9 ± 0.1
Yb 0.1%	3.7844 ± 0.0002	9.4984 ± 0.0005	27.1 ± 0.2
Yb 0.2%	3.7848 ± 0.0003	9.4997 ± 0.0008	27.6 ± 0.2
Yb 0.3%	3.7849 ± 0.0002	9.4986 ± 0.0005	25.9 ± 0.1

**Table 2:** Specific surface area values of the for the 0.2% metal atoms solid solution samples.

Sample	Specific surface area/m^2^ g^−1^
Pr 0.2%	76.3 ± 0.3
Nd 0.2%	60.1 ± 0.3
Sm 0.2%	73.9 ± 0.3
Gd 0.2%	72.2 ± 0.3
Er 0.2%	105.4 ± 0.3
Yb 0.2%	84.4 ± 0.3

**Table 3:** Photoanode thickness and dye loading values for the all the solid solutions.

Sample	*Photoanode thickness/μm*	Dye loading/mg*cm^*−2*^
Pr 0.1%	12 ± 1	0.128 ± 0.001
Pr 0.2%	13 ± 1	0.163 ± 0.001
Pr 0.3%	14 ± 1	0.136 ± 0.001
Nd 0.1%	11 ± 1	0.160 ± 0.001
Nd 0.2%	12 ± 1	0.092 ± 0.001
Nd 0.3%	11 ± 1	0.106 ± 0.001
Sm 0.1%	12 ± 1	0.143 ± 0.001
Sm 0.2%	14 ± 1	0.126 ± 0.001
Sm 0.3%	12 ± 1	0.138 ± 0.001
Gd 0.1%	13 ± 1	0.156 ± 0.001
Gd 0.2%	11 ± 1	0.079 ± 0.001
Gd 0.3%	12 ± 1	0.157 ± 0.001
Er 0.1%	12 ± 1	0.099 ± 0.001
Er 0.2%	11 ± 1	0.093 ± 0.001
Er 0.3%	14 ± 1	0.125 ± 0.001
Yb 0.1%	13 ± 1	0.080 ± 0.001
Yb 0.2%	13 ± 1	0.122 ± 0.001
Yb 0.3%	13 ± 1	0.098 ± 0.001

**Table 4:** In the second column, the band gap values of the samples under study extrapolated from the Tauc plots of the Kubelka-Munk function calculated for the indirect interband transition.

Sample	Band gap/eV	*J*_*SC*_/mA cm^−2^	*V*_*OC*_/V	*η*/%	*FF*	*R*_*S*_/Ω	*J*_0_/nA cm^−2^	*m*	*V*_*OC*_*calc/*V
TiO_2_	3.269 ± 0.002	13.9 ± 0.1	0.7155 ± 0.0001	7.0 ± 0.1	0.70 ± 0.01	88 ± 1	0.73 ± 0.03	1.31 ± 0.01	0.564 ± 0.001
Pr 0.1%	3.268 ± 0.002	12.4 ± 0.1	0.7021 ± 0.0001	6.2 ± 0.1	0.72 ± 0.01	88 ± 1	4.9 ± 0.5	2.03 ± 0.01	0.768 ± 0.001
Pr 0.2%	3.273 ± 0.002	14.4 ± 0.1	0.6888 ± 0.0001	6.8 ± 0.1	0.69 ± 0.01	74 ± 1	14.4 ± 0.6	2.09 ± 0.01	0.762 ± 0.001
Pr 0.3%	3.244 ± 0.004	10.8 ± 0.1	0.7303 ± 0.0001	5.7 ± 0.1	0.72 ± 0.01	101 ± 1	5.0 ± 0.5	1.95 ± 0.02	0.732 ± 0.001
Nd 0.1%	3.291 ± 0.001	12.7 ± 0.1	0.7387 ± 0.0001	6.7 ± 0.1	0.72 ± 0.01	76 ± 1	50 ± 1	2.52 ± 0.01	0.807 ± 0.001
Nd 0.2%	3.244 ± 0.002	13.3 ± 0.1	0.7319 ± 0.0001	6.9 ± 0.1	0.71 ± 0.01	98 ± 1	5.6 ± 0.7	1.91 ± 0.02	0.719 ± 0.001
Nd 0.3%	3.256 ± 0.002	12.7 ± 0.1	0.7462 ± 0.0001	6.8 ± 0.1	0.71 ± 0.01	81 ± 1	27 ± 2	2.45 ± 0.01	0.822 ± 0.001
Sm 0.1%	3.283 ± 0.002	15.2 ± 0.1	0.6964 ± 0.0001	7.5 ± 0.1	0.71 ± 0.01	66 ± 1	5.3 ± 0.3	2.00 ± 0.01	0.764 ± 0.001
Sm 0.2%	3.273 ± 0.002	15.6 ± 0.1	0.7317 ± 0.0001	8.2 ± 0.1	0.72 ± 0.01	71 ± 1	13 ± 1	2.09 ± 0.02	0.752 ± 0.001
Sm 0.3%	3.271 ± 0.002	14.9 ± 0.1	0.6878 ± 0.0001	7.3 ± 0.1	0.71 ± 0.01	68 ± 1	5.6 ± 0.4	2.00 ± 0.01	0.760 ± 0.001
Gd 0.1%	3.299 ± 0.002	14.7 ± 0.1	0.6975 ± 0.0001	7.2 ± 0.1	0.70 ± 0.01	68 ± 1	6.5 ± 0.8	2.07 ± 0.02	0.777 ± 0.001
Gd 0.2%	3.287 ± 0.001	15.5 ± 0.1	0.7127 ± 0.0001	7.9 ± 0.1	0.72 ± 0.01	64 ± 1	74 ± 2	2.56 ± 0.01	0.806 ± 0.001
Gd 0.3%	3.289 ± 0.001	13.9 ± 0.1	0.7078 ± 0.0001	6.8 ± 0.1	0.69 ± 0.01	75 ± 1	5.4 ± 0.3	2.06 ± 0.01	0.783 ± 0.001
Er 0.1%	3.217 ± 0.002	13.8 ± 0.1	0.7350 ± 0.0001	7.4 ± 0.1	0.73 ± 0.01	78 ± 1	0.57 ± 0.06	1.66 ± 0.01	0.726 ± 0.001
Er 0.2%	3.265 ± 0.002	16.7 ± 0.1	0.7313 ± 0.0001	8.7 ± 0.1	0.71 ± 0.01	65 ± 1	1.5 ± 0.2	1.57 ± 0.01	0.655 ± 0.001
Er 0.3%	3.176 ± 0.002	14.9 ± 0.1	0.7265 ± 0.0001	7.6 ± 0.1	0.70 ± 0.01	78 ± 1	4.0 ± 0.6	1.90 ± 0.02	0.740 ± 0.001
Yb 0.1%	3.274 ± 0.001	13.7 ± 0.1	0.7648 ± 0.0001	7.2 ± 0.1	0.69 ± 0.01	89 ± 1	0.63 ± 0.08	1.70 ± 0.02	0.738 ± 0.001
Yb 0.2%	3.266 ± 0.002	16.0 ± 0.1	0.7422 ± 0.0001	8.4 ± 0.1	0.71 ± 0.01	72 ± 1	0.29 ± 0.06	1.47 ± 0.06	0.672 ± 0.001
Yb 0.3%	3.277 ± 0.002	12.0 ± 0.1	0.7534 ± 0.0001	6.5 ± 0.1	0.72 ± 0.01	101 ± 1	0.9 ± 0.1	1.73 ± 0.02	0.733 ± 0.001

In the following columns, DSSC parameters extrapolated from *J*–*V* curves under irradiation and in the dark. *J*_*SC*_, *V*_*OC*_, *η*, *FF* and *R*_*S*_ were obtained from curves under illumination, while *J*_0,_ *m* and *V*_*OC*_ *calc* the curves in dark using the modified diode equations.

**Table 5:** Electron diffusion length and charge collection efficiency values obtained by the fit of EIS spectra using the transmission line model.

Sample	*Electron diffusion length/μm*	*Collection efficiency*
Pr 0.1%	51 ± 4	0.948 ± 0.001
Pr 0.2%	43 ± 4	0.915 ± 0.001
Pr 0.3%	70 ± 4	0.961 ± 0.001
Nd 0.1%	52 ± 4	0.958 ± 0.001
Nd 0.2%	47 ± 4	0.939 ± 0.001
Nd 0.3%	46 ± 4	0.946 ± 0.001
Sm 0.1%	61 ± 4	0.963 ± 0.001
Sm 0.2%	47 ± 4	0.920 ± 0.001
Sm 0.3%	55 ± 4	0.955 ± 0.001
Gd 0.1%	44 ± 4	0.920 ± 0.001
Gd 0.2%	46 ± 4	0.947 ± 0.001
Gd 0.3%	46 ± 4	0.937 ± 0.001
Er 0.1%	97 ± 4	0.985 ± 0.001
Er 0.2%	94 ± 4	0.986 ± 0.001
Er 0.3%	66 ± 4	0.956 ± 0.001
Yb 0.1%	110 ± 4	0.986 ± 0.001
Yb 0.2%	122 ± 4	0.989 ± 0.001
Yb 0.3%	93 ± 4	0.981 ± 0.001
